# Feeding-fasting dependent recruitment of membrane microdomain proteins to lipid droplets purified from the liver

**DOI:** 10.1371/journal.pone.0183022

**Published:** 2017-08-11

**Authors:** Kritika Sadh, Priyanka Rai, Roop Mallik

**Affiliations:** Department of Biological Sciences, Tata Institute of Fundamental Research, Mumbai, India; University of British Columbia, CANADA

## Abstract

Lipid droplets (LDs) are cellular stores of neutral fat that facilitate lipid and protein trafficking in response to metabolic cues. Unlike other vesicles, the phospholipid membrane on the LD is a monolayer. Interestingly, this monolayer membrane has free cholesterol, and may therefore contain lipid microdomains that serve as a platform for assembling proteins involved in signal transduction, cell polarity, pathogen entry etc. In support of this, cell culture studies have detected microdomain-associated “raftophilic” proteins on LDs. However, the physiological significance of this observation has been unclear. Here we show that two proteins (Flotillin-1 and SNAP23) that bind to membrane microdomains associate differently with LDs purified from rat liver depending on the feeding/fasting state of the animal. Flotillin-1 increases on LDs in the fed state, possibly because LDs interact with the endoplasmic reticulum (ER), facilitating supply of flotillin-1 from ER to LDs. Interestingly, this increase in flotillin-1 is correlated with an increase in free cholesterol on the LDs in fed state. In opposite behaviour to flotillin-1, SNAP23 increases on LDs in the fasted state and this appears to mediate LD-mitochondria interactions. Such LD-mitochondria interactions may provide fatty acids to mitochondria for promoting beta-oxidation in hepatocytes in response to fasting. Our work brings out physiologically relevant aspects of lipid droplet biology that are different from, and may not be entirely possible to replicate and study in cell culture.

## Introduction

Lipid droplets (LDs) are fat storing organelles commonly found in prokaryotic and eukaryotic cells. LDs have a hydrophobic core containing triglyceride (TG) and esterified cholesterol, surrounded by a phospholipid monolayer where a variety of proteins are embedded [1]. These organelles are primarily involved in regulated storage and release of neutral lipids, however protein sequestration and transport are also well-known functions of LDs [2,3]. Altered molecular composition of LDs can trigger significant remodelling of the LD membrane [4], and this is relevant to several metabolic pathologies [5]. Interestingly, the monolayer phospholipid membrane on LDs also contains free cholesterol (FC), a molecule that aids formation of membrane microdomains in the plasma membrane [1,6]. These microdomains are suspected platforms for assembly of proteins involved in signal transduction, cell polarity, pathogen entry etc. [7].

Lipid microdomains are segregated by their compactness from the neighbouring membrane [8], and could be planar (lipid rafts) or invaginations (caveolae). “Raftophilic” proteins known to associate with lipid microdomains have been found on LDs [9–11]. While the existence of caveolae on LDs is much discussed [12,13], the occurrence of planar rafts on LDs has not been addressed. Lipid rafts were detected by Raman imaging on artificial monolayer membranes [14], and therefore rafts may be present on LDs. Supporting this possibility, a palmitoylated form of the protein ELMOD2 can bind to LDs [15]. Since pamitoylation favours binding of proteins to cholesterol rich microdomains [8], ELMOD2 may bind to microdomains on the LD membrane. Further supporting the existence of microdomains on LDs, the cholesterol binding protein flotillin-1 was detected on LD membranes [16]. Flotillin-1 belongs to the SPFH family of lipid raft binding proteins [17]. Flotillins have generated significant interest because of their roles in signal transduction, cholesterol homeostasis, cell adhesion, T cell receptor signalling and clathrin independent endocytosis [18]. Flotillin localises dynamically to subcellular compartments, and signalling molecules can induce it’s redistribution from plasma membrane (PM) to endosomes [19]. Cell culture studies show that flotillin-1 localises to the LD membrane in response to oleic acid (OA) treatment. Fusion of LDs with the PM can lead to exocytosis of flotillin-1 from LDs [10,20]. LDs have also been implicated in numerous flotillin-1 related pathways, for example insulin signalling and cholesterol transport [21,22]. Another microdomain associated protein, SNAP23 is also present on the LD membrane as a part of the SNARE complex [23,24]. SNAP23 facilitates transient fusion events between LDs and mitochondria, likely promoting fatty acid transfer to mitochondria for beta oxidation [25].

While the above reports bring out interesting possible functions for microdomain-associated proteins on LDs, all of them use cultured cells. The physiological relevance for appearance of microdomain-associated proteins on LDs is unknown. To address this issue, we investigated the association of flotillin-1 and SNAP23 with LDs purified from rat liver in fed and fasted state of the animal. Fasting accentuates the role of liver as a metabolic homeostat that protects the body from lipid-induced disorders. During fasting, rapid TG breakdown is initiated in other tissues (mainly adipose), thus increasing free fatty acid (FFA) in circulation. This FFA is delivered to the liver, where it is stored in the form of TG in LDs of hepatocytes, followed by release of TG into circulation in the form of lipoprotein (VLDL) particles [26,27]. These functions of the liver, namely storage and controlled release of TG, buffer other organs against lipotoxic effects of circulating FFA. How specific proteins are recruited to LDs across feeding/fasting transitions, and it’s implication for lipid/protein flux in the animal is important.

We find here that flotillin-1 association with LDs is higher in the liver of fed rats as compared to fasted, and a similar observation is made for free cholesterol (FC) levels on the LDs. The flotillin-1 level on ER-enriched microsomes shows an opposite trend, suggesting that flotillin-1 is trafficked from ER to LDs in fed state, but this trafficking is inhibited upon fasting. Interestingly, SNAP23 recruitment to LDs shows the opposite behaviour to flotillin-1, with more SNAP23 recruited to LDs in fasted state. This trend is also found to be true for mitochondrial marker proteins, suggesting that SNAP23 engineers increased LD-mitochondria interactions. Such interactions may provide mitochondria with FA required for beta-oxidation in hepatocytes in fasted state. In support of this, LD-mitochondria interactions in response to starvation are widely reported in cell culture models [25,28,29].

## Results

### Purification of lipid droplets from liver of fed and fasted rats

**Fig 1:**
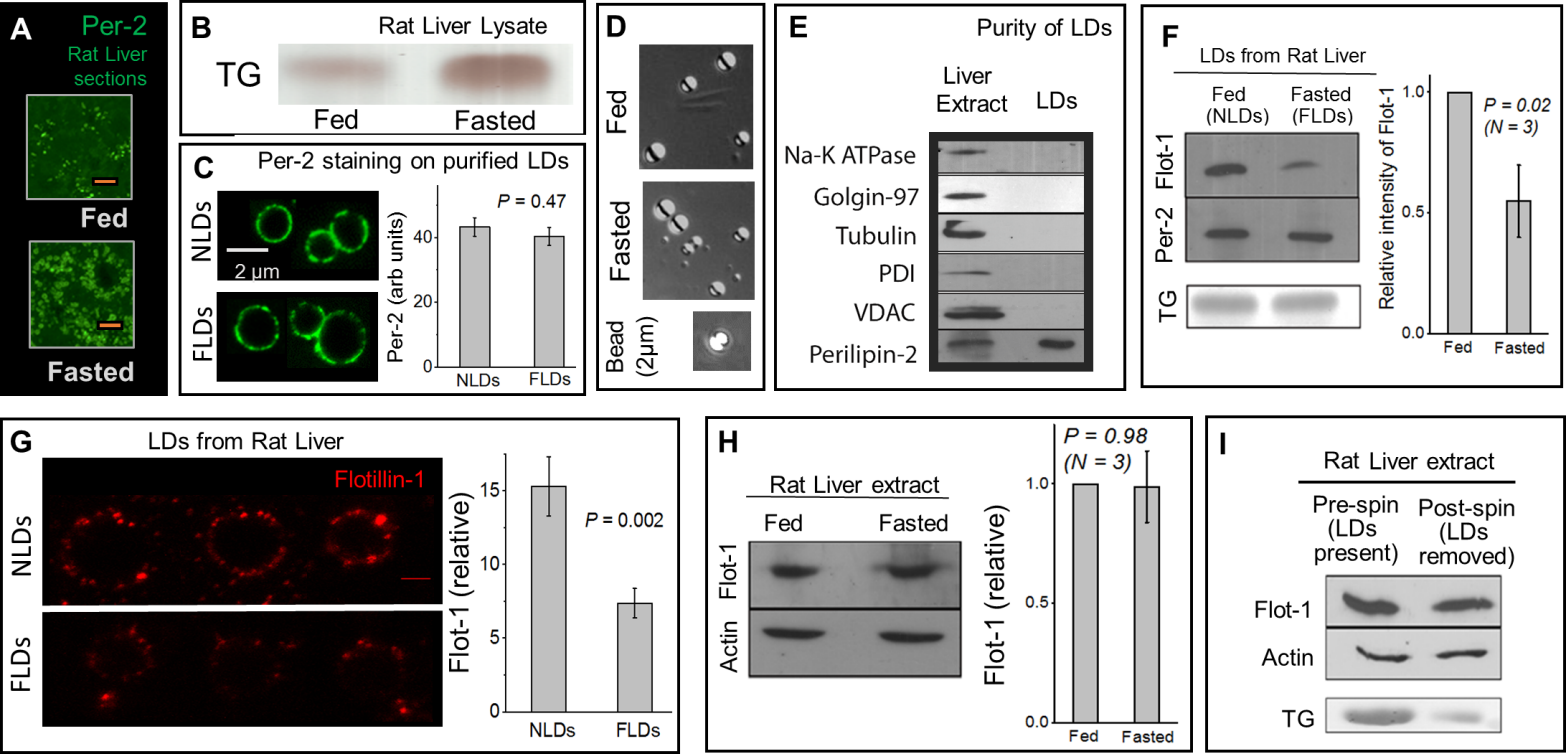
Flotillin-1 binding to LDs purified from liver is reduced in fasted state. (A) Immunostaining of LDs using anti-perilipin-2 antibody on liver tissue sections from fed and fasted rats. There is a drastic increase in number of LDs after 16 hours of fasting. Scale bar = 10μm. (B) TLC showing significantly higher TG levels in rat liver lysate after 16 hours of fasting. (C) Staining of purified NLDs and FLDs using a perilipin-2 antibody. There is no difference in the amount of perilipin-2 on NLDs and FLDs at the single-LD level. Panel on right quantifies the changes. 30 LDs used per condition. Error bars are SEM. (D) DIC image of LDs purified from liver in fed and fasted state. A 2 μm latex bead is also shown. There is no significant change in the size of purified LDs between fed and fasted state (see main text). (E) The LD fraction isolated from liver is free of detectable contamination from PM (Na-K ATPase used as marker), Golgi (Golgin-97), cytosolic (tubulin), ER (PDI) and mitochondrial (VDAC-1). Liver extract is loaded as a positive control. The LD fraction is enriched in perilipin-2 (LD marker). (F) Flotillin-1 is detected on LDs isolated from liver of normally fed rats (NLDs) and fasted rats (FLDs) by Western blotting. Flotillin-1 levels reduce on LDs upon fasting. Equal loading of NLDs and FLDs is confirmed from equal perilipin-2 levels (by Western blotting) and equal TG (by TLC) in these samples. Right panel quantifies the changes. Error bars are SEM. (G) Flotillin-1 staining on isolated NLDs and FLDs. Representative LDs of comparable size from each sample are shown. Scale bar is 2μm. Right panel shows the flotillin-1 fluorescence intensity (mean±sem) measured along circumference of individual NLDs and FLDs (15 of each used). (H) Flotillin-1 level does not change significantly in liver tissue extract between fed and fasted state. This observation was consistent in samples collected from three pairs of animals. Western blotting against actin shows equal loading of the liver extract samples. Error bars are SEM (I) Western blotting against flotillin-1 of liver extract from fasted rat before and after depletion of LDs by centrifugation. No detectable reduction is seen in flotillin-1 levels after removal of LDs (post-spin). Western blotting of actin shows equal loading of the TE samples. Depletion of LDs from the liver extract is confirmed by reduction in TG levels through TLC.

As a result of fasting, the body switches from glucose to TG utilization. FAs released from adipose reserves reach the liver, are converted into TG, and re-stored as LDs in hepatocytes. This accumulates LDs massively in the fasted liver [30–32]. Such an increase was confirmed by immunohistochemical (IHC) analysis (Fig 1A) of rat liver sections using an antibody against perilipin-2 (LD marker protein). Thin layer chromatography (TLC) of liver tissue from fed and fasted rats also revealed massive increase in TG after fasting (Fig 1B). This increase was 3.4±0.4 fold (mean±sem; 3 pairs of animals tested), and is in good agreement with other reports [32].

We have described a detailed protocol (also see methods) for the purification of LDs from rat liver by density gradient ultracentrifugation [33,34]. LDs purified from liver of normally fed rats will be called **N****ormal** **LD**s **(NLDs)**. LDs purified from liver of 16 hour fasted rats will be called **F****asted** **LD**s **(FLDs).** Immunofluorescence imaging using an antibody against perilipin-2 on purified NLDs and FLDs showed robust staining along the circumference (Fig 1C; see Methods), suggesting that there is no gross disruption of the LD membrane during purification. Quantification of fluorescence intensity along LD circumference also suggested no significant change in perilipin-2 amount between NLDs and FLDs (Fig 1C; 30 LDs used per condition).

Fig 1D shows differential interference contrast images of NLDs and FLDs along with a latex bead of 2 micron diameter. We observed no significant difference in size between NLDs and FLDs purified from multiple animals [Diameter of NLDs = 1.96 ± 0.76μm (173 LDs used); FLDs = 1.90 ± 0.80μm (178 LDs used); *P* = 0.47]. Thus, there are more LDs in the liver after fasting, but the size of individual purified LDs does not change. Perilipin-2 staining of LDs in liver sections also suggested no obvious differences in LD size (Fig 1A). Western blotting to test the purity of LDs showed that the isolated LD fraction was enriched in the LD marker protein perilipin-2, with no detectable presence of cytosolic, plasma membrane, mitochondria, ER and Golgi contamination (Fig 1E). This is expected because the LDs are highly buoyant and float to the top in a sucrose gradient [33,34].

Here we wanted to compare protein levels on LDs in fed/fasted states using biochemical assays such as western blotting. We therefore invested significant effort in developing a method to ensure equal loading of two LD samples (for example, an NLD sample and an FLD sample). We did not assume *a-priori* that NLDs and FLDs have the same amount of (total) protein on their surface because fasting could induce changes in the lipidome and proteome of the LDs. Rather, we used a procedure for equal loading based on the scattering of light from refractile LDs (Methods). We have used this procedure extensively for equal loading of latex bead phagosome samples [35]. These latex bead phagosomes are of similar size (few microns in diameter) and refractive index as LDs purified from rat liver (compare images of LDs with latex bead in Fig 1D). This method was checked by observing a linear variation in the optical density at 400nm (OD_400_) of serially diluted purified LDs (not shown). Purified NLD and FLD samples were therefore appropriately diluted to obtain equal OD_400_. After completing this procedure, reassuringly, we found that equal volumes of these samples (presumably having equal number of LDs) do have equal amounts of total TG and protein. We therefore concluded that NLD and FLD samples can also be normalized by total protein.

### Feeding-fasting dependent association of flotillin-1 with LDs in the liver

Flotillin-1, a lipid raft associated marker protein, has been found on LDs in several cell culture studies [10,16,20]. We asked if association of flotillin-1 with hepatic LDs is correlated with the feeding/fasting state. Western blotting detected abundant flotillin-1 on NLDs, but flotillin-1 levels were lower on FLDs across multiple preparations (Fig 1F). The amount of perilipin-2 as well as TG was same on NLD and FLD samples (Fig 1F), confirming that equal LDs were loaded in both cases. To verify at the single-LD level that flotillin-1 decreases after fasting, immunostaining was performed on purified NLDs and FLDs (Fig 1G) using a microchamber immunofluorescence assay (Methods). In agreement with the Western blotting results, mean flotillin-1 intensity along the LD circumference was approximately two-fold higher on individual NLDs as compared to FLDs (Fig 1G, right panel). We found it difficult to image flotillin-1, and to quantify changes of flotillin-1 on LDs in liver sections because of background staining and the abundance of LDs in fasted liver.

We next compared flotillin-1 levels in tissue extract (TE) prepared from liver of fed and fasted rats to find no significant difference (Fig 1H). Availability of flotillin-1 to LDs could become limited in the fasted state because the total flotillin-1 in liver is same (Fig 1H), but the number of LDs is increased after fasting. This may trivially explain the reduction of flotillin-1 on FLDs. To investigate if this is true, we prepared liver extract from a fasted rat and divided it into two equal parts. One part was retained (pre-spin; contains LDs in the sample), and the other part was centrifuged to remove the buoyant LDs (post-spin). Removal of LDs was confirmed by observing lower levels of TG in the post-spin sample (Fig 1I). Western blotting of these samples showed no observable reduction of flotillin-1 in the post-spin sample as compared to pre-spin (Fig 1I). This shows that the LD-bound flotillin-1 that was removed by centrifugation of LDs is a negligible fraction of the total flotillin-1 present in liver extract. Therefore, availability of flotillin-1 to LDs is not limiting in the fasted liver. A similar inference has also been reported for stomatins [9]. Therefore, the reduction of flotillin-1 on FLDs likely arises from specific metabolic pathways that control recruitment of flotillin-1 to LDs in feeding-fasting dependent manner.

### Flotillin-1 may be supplied to LDs from the ER

**Fig 2:**
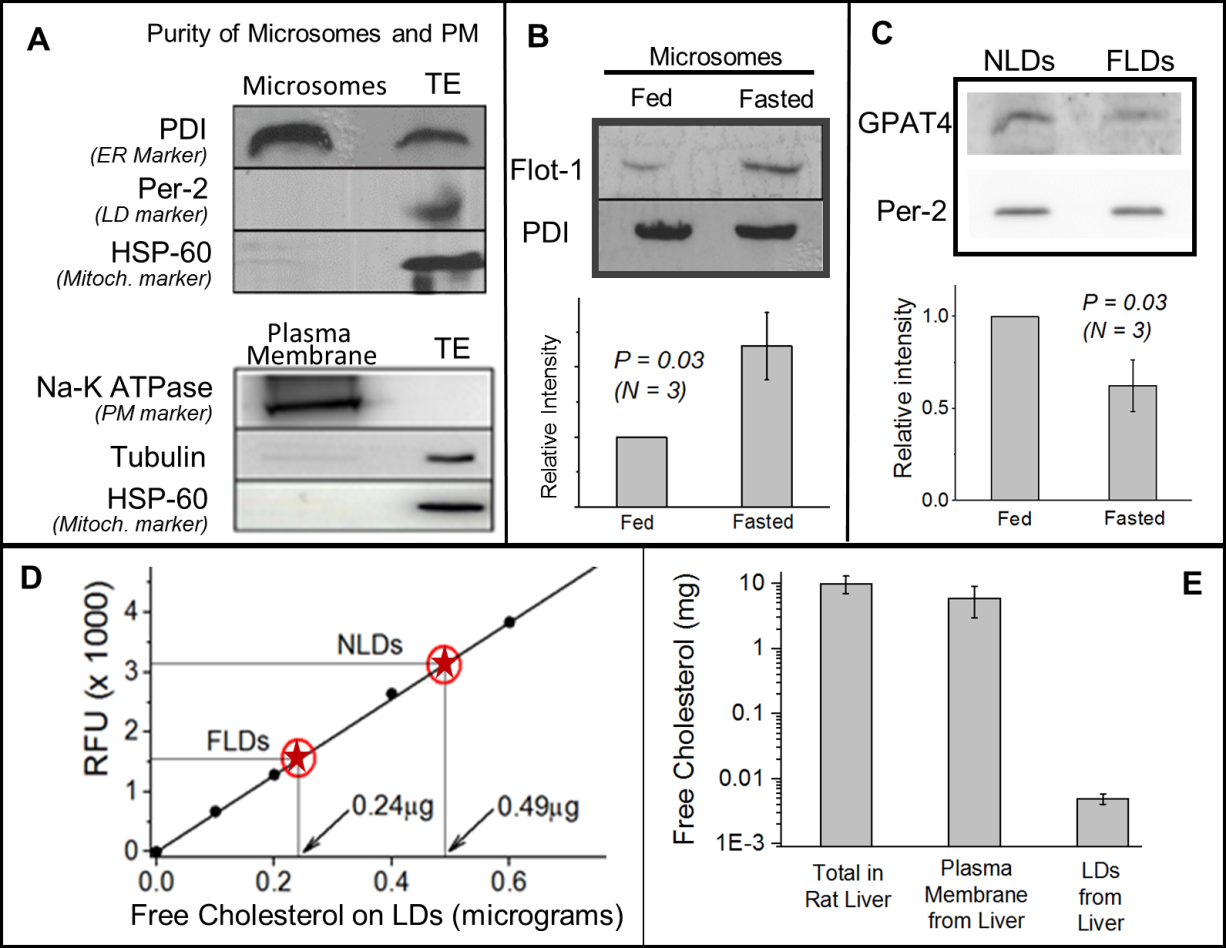
Feeding-fasting dependent changes in flotillin-1, GPAT4 and free cholesterol (FC) on LDs and ER-enriched microsomes. (A) Upper panel The microsome fraction isolated from normally fed rat liver is enriched in PDI (ER marker). Mitochondrial marker (HSP-60) and LD marker (perilipin-2) are not detected on the microsomes. Liver tissue extract (TE) with equal amount of protein as microsomes was loaded. Lower panel A membrane fraction isolated from normally fed liver is enriched in the plasma membrane marker (Na-K ATPase). No cytosolic or mitochondrial contamination is detected. (B) Flotillin-1 levels increase on the microsome fraction in fasted liver as compared to fed state. Western blotting against PDI (ER marker) shows equal loading of the ER samples. This experiment was repeated with consistent results on three pairs of fed and fasted animals. Error bars are SEM. (C) GPAT4 localisation on LDs decreases after fasting. Western blotting using anti-GPAT4 antibody shows higher level on NLDs as compared to FLDs. Equal amount of NLD and FLD samples was loaded, as determined from equal intensity of perilipin-2 band on these samples. This experiment was repeated on three pairs of fed and fasted animals, yielding similar results. Error bars are SEM. (D) Measurement of free cholesterol (FC) by a fluorimetric method. Y-axis denotes relative fluorescence units (RFU). Known amounts of cholesterol standards (filled circles) were used to build a calibration curve (straight line). The RFU value was then measured for NLDs and FLDs (red circles with star). The corresponding amount of FC in these samples was then read off from the calibration curve (shown in graph). This measurement shows two-fold more FC in NLDs as compared to FLDs. Equal NLD and FLD samples were loaded, as determined from equal TG amounts in the samples in TLC experiments. Duplicates of all the samples were used for the assay. This experiment was repeated twice with similar results (using two pairs of animals). (E) Estimation of free cholesterol in whole rat liver tissue extract, plasma membranes from rat liver, and LDs purified from rat liver. Liver of normally fed rats were used. Note the logarithmic scale on Y axis. Crude liver tissue extract sample was prepared from a known mass of liver tissue, and a TLC experiment was run with this sample, along with known dilutions of FC as standards (not shown). The best match was used to estimate total amount of FC in the liver extract sample (*N* = 3). Similarly, PM and LDs were prepared and comparison was done with FC standards in a TLC experiment. The total FC on PM and LDs was then estimated for the whole liver (see methods).

We next probed how flotillin-1 might be trafficked to LDs. Flotillin-1 shares common structural and membrane-binding properties with stomatins and caveolins [10,36], which are trafficked to LDs from ER [9,37]. To investigate if a similar pathway is operative for flotillin, we isolated microsomes from liver of fed and fasted rats (see Methods). This microsome fraction was enriched in the ER-marker protein PDI, but LD and mitochondrial markers were below detectable levels (Fig 2A; top panel). Only results for ER-enrichment on microsomes prepared from liver of a fed rat are shown. Similar ER-enrichment was also observed for fasted rats (not shown). Significantly less flotillin-1 was detected on microsomes obtained from liver of fed animals as compared to fasted (Fig 2B). A Western blot for PDI (ER marker) confirmed that equal amount of protein was loaded in the fed and fasted microsome samples. This observation is opposite to LDs (more flotillin-1 on LDs from fed liver; Fig 1F), and suggests that flotillin-1 is retained in the ER in fasted state, blocking its transport to LDs.

We hypothesized that transport of flotillin-1 from ER to LDs is inhibited in fasted state because fasting reduces physical interactions between ER and LDs. If this is true, fasting should also inhibit trafficking of other proteins from ER to LDs. Glycerol-3-phosphate acyltransferase 4 (GPAT4) is a widely studied enzyme that catalyzes formation of TG on LDs. GPAT4 relocalizes from the ER to LDs at physical contacts between these two organelles. This ER-to-LD targeting of GPAT4 leads to TG biosynthesis on the LD and increase in LD size [38]. Comparison of the levels of GPAT4 showed a consistent reduction on FLDs as compared to NLDs across three pairs of animals (Fig 2C). This reduction of GPAT4 is concomitant with reduction of flotillin-1 on FLDs, and suggests that ER-LD interactions may be downregulated in hepatocytes in fasted state. Notably, the transfer of GPAT4 was shown to be unidirectional from ER to LDs, and once recruited to LDs, GPAT4 did not leave the LDs [38]. We therefore interpret our data as enhanced transfer of flotillin-1 and GPAT4 from ER-to-LDs because of ER-LD interactions in fed state. The scenario of reverse (LD-to-ER) trafficking in fasted state, while formally possible, appears unlikely.

### Flotillin-1 targeting to LDs is correlated with free cholesterol levels

Flotillin-1 is known to bind free cholesterol (FC) on lipid membranes, and is also involved in trafficking of cholesterol in response to external stimuli [17]. To understand the cause of flotillin-1 association with LDs, we therefore compared FC levels between NLDs and FLDs. Total lipids were extracted from LD samples and FC was measured using a sensitive cholesterol estimation kit (Methods). Known amounts of cholesterol standards were used to build a calibration curve. This calibration curve is shown in Fig 2D by black circles fitted with a straight line. We observed two-fold enrichment of FC on NLDs as compared to FLDs (Fig 2D; NLD and FLD data marked with red stars). Equal TG content (as a loading control) was confirmed by TLC for both the LD samples (not shown). While we do observe a difference in FC between NLDs and FLDs, it may be noted that the absolute amounts of FC on LDs (micrograms) is quite low when compared to other cellular organelles (see below).

**Fig 3:**
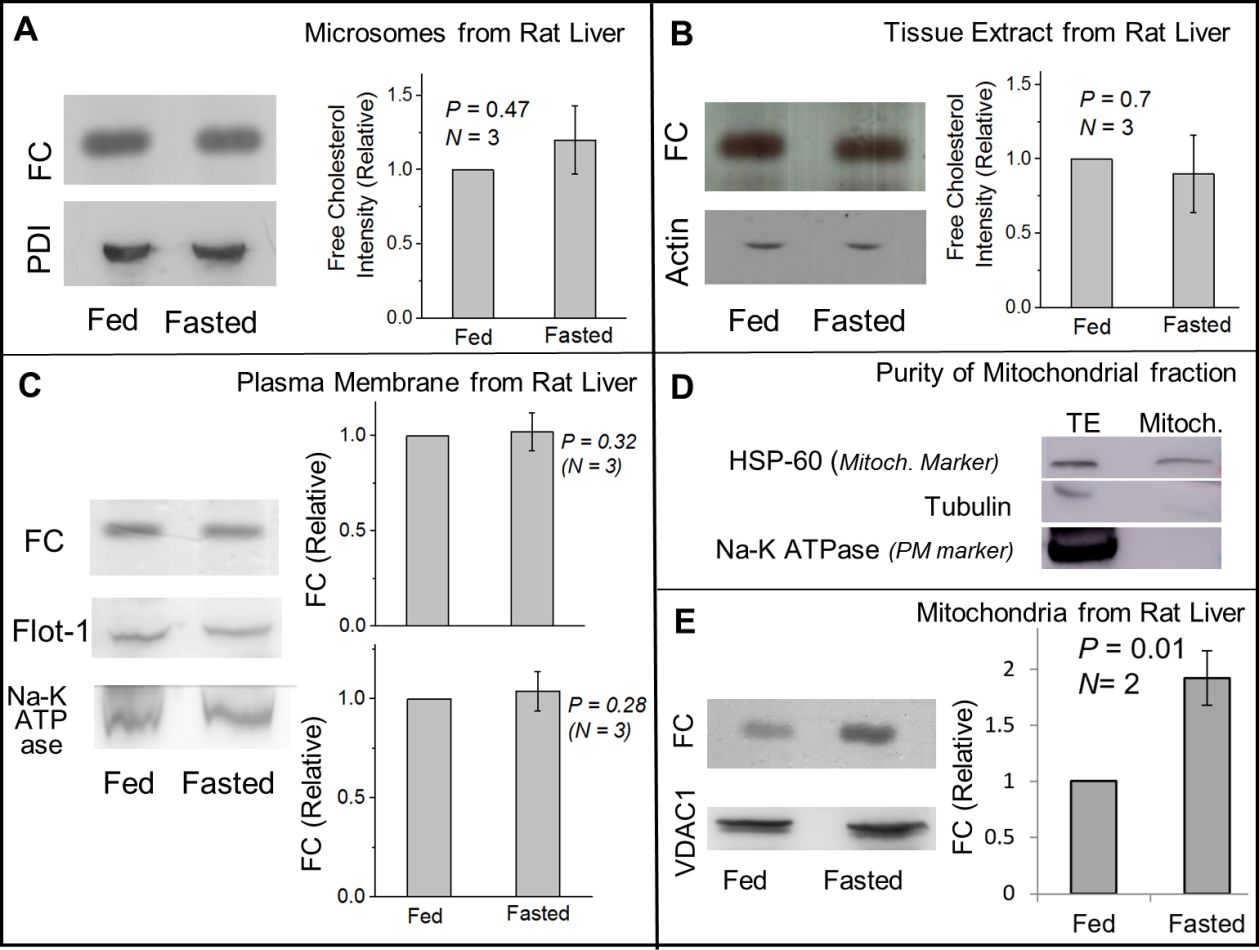
Comparison of free cholesterol levels between fed and fasted states on ER-enriched microsomes, liver tissue extract, plasma membrane and mitochondria. (A) TLC showing equal FC levels in microsomes isolated from liver of fed and fasted rats. Equal loading was confirmed by western blotting for the ER marker PDI. Statistical analysis shows no significant change in FC levels in ER after fasting. Error bars are SEM. (B) TLC showing equal FC levels in total liver lysate prepared from fed and fasted rats. Western blotting for actin shows equal loading of liver lysate samples. Error bars are SEM. (C) TLC showing equal FC levels in PM-enriched membrane fractions isolated from liver of fed and fasted rats. Flotillin-1 also shows no change between these states. Western blotting for Na-K ATPase (PM marker) confirms equal loading of samples. There is no statistical difference in FC or Flot-1 levels on PM between fed and fasted liver. Error bars are SEM. (D) To determine the purity of mitochondria fraction, equal amount of protein of liver tissue extract (TE) and mitochondria (Mitoch.) were loaded in a gel for western blotting. The fraction was enriched in mitochondrial marker protein HSP60. Cytosolic (tubulin) and plasma membrane (Na-K ATPase) markers could not be detected in this fraction. (E) TLC on liver mitochondria samples shows higher FC levels in fasted as compared to fed state. Samples were normalised for equal protein, as confirmed by presence of VDAC1 (mitochondria marker). A statistically significant increase is observed in FC in fasted state as compared to fed state.

Since flotillin-1 can bind FC [17], it is possible that trafficking of FC and flotillin-1 from ER to LDs is interconnected. Before attempting to address this question, we point out that the FC amount on LDs is four orders of magnitude less than in whole liver tissue and PM (Fig 2E; also see methods). Therefore, even if FC trafficks between LDs and one/more of these organelles, this may not significantly change the FC on the organelle. Indeed, the reduction in FC on LDs in fasted state (Fig 2D) was not accompanied by any detectable increase in FC on rat liver microsomes (Fig 3A), liver tissue extract (Fig 3B) and plasma membrane (PM) from rat liver (Fig 3C). Since the PM is intimately associated with FC trafficking and homeostasis [22], we also checked flotillin-1 levels in a PM enriched fraction from rat liver. Enrichment of PM markers was confirmed in this fraction (Fig 2A; lower panel). However, no change in flotillin-1 on PM could be detected between fed and fasted states (Fig 3C), likely because of the small amount of flotillin-1 on LDs (relative to PM).

Because LDs interact and exchange FA with mitochondria, we also investigated changes of FC in a purified mitochondrial fraction from fed and fasted rat liver. The mitochondria marker HSP-60 was enriched in this mitochondrial fraction, but cytosolic and PM markers could not be detected (Fig 3D). Significantly more FC was observed in the mitochondrial fraction from liver of fasted rat (Fig 3E). However, the FC amount on mitochondria is known to be much higher [39] than the microgram amounts that we detected on LDs. Therefore, our study cannot infer that LDs exchange FC with mitochondria. It is likely that FC is trafficked to mitochondria from other cellular organelles in the fasted state. However, the results on flotillin-1 (Fig 1F and Fig 2B) and GPAT4 (Fig 2C) suggest enhanced ER-LD interactions as a cause for FC and flotillin-1 trafficking to LDs in fed state. In support of this possibility, cholesterol is reported to induce ER-LD interactions in cultured hepatocytes [40]. FC levels are tightly regulated in the ER because perturbations can lead to apoptotic response [41], and this could be why FC in the microsome fraction remains unchanged even if FC is trafficked between the ER and LDs. If the amount of FC on LDs is so small (micrograms; Fig 2D), can relative changes in FC between fed and fasted states impact the cell biology of LDs? This question remains to be answered fully, but the existing literature does suggest this possibility [7,10,11].

### SNAP23 recruitment to LDs shows opposite behaviour compared to flotillin-1

**Fig 4:**
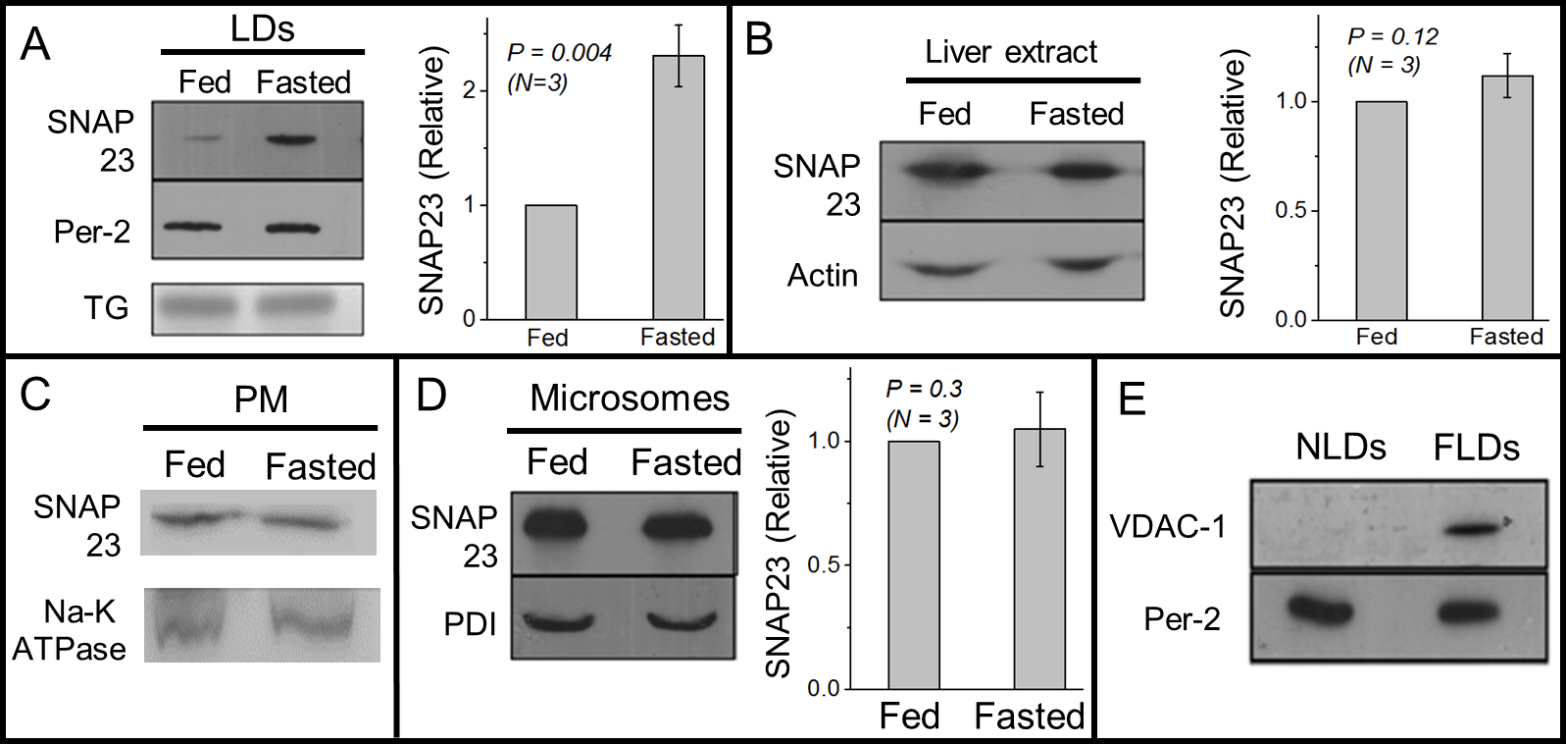
SNAP23 association with LDs, plasma membrane and microsomes across feeding-fasting transition. (A) Western blotting against SNAP23 on NLD and FLD samples shows more SNAP23 on FLDs as compared to NLDs. Equal amount of NLDs and FLDs were used, as confirmed from equal intensity of perilipin-2 band by Western blotting and by TG band intensity in a TLC experiment. The experiment was repeated on three pairs of fed and fasted animals. Relative intensity of SNAP23 normalised to perilipin-2 levels has been used for statistical analysis. Error bars are SEM. (B) SNAP23 levels do not show significant change in liver extract prepared from fed and fasted rats. Actin was used as loading control. This observation was seen consistently in samples prepared from three pairs of animals. Error bars are SEM. (C) No significant change is observed in SNAP23 levels on PM-enriched fractions between fed and fasted states. Western blotting against Na-K ATPase (PM marker) was done to ensure equal loading of samples. (D) No significant change is observed in SNAP23 levels on microsome fractions between fed and fasted states. Western blotting against PDI shows equal loading of both the samples. Error bars are SEM. (E) Western blotting against VDAC-1 (mitochondria marker) on NLD and FLD samples. VDAC-1 is detected only on FLDs. This experiment was repeated on LD samples isolated from two pairs of fed and fasted animals.

We next asked if fasting leads to reduced association of another microdomain associated protein SNAP23 to the LDs. Opposite to flotillin-1, SNAP23 levels were significantly higher on FLDs as compared to NLDs (Fig 4A). SNAP23 levels did not change significantly between liver extracts from fed and fasted rats (Fig 4B). We had earlier ruled out the possibility that flotillin-1 availability to LDs becomes limiting in the fasted state when LD numbers increase after fasting (see Fig 1I). We did not repeat this test for SNAP23 because there are lesser LDs in the fed state in liver, and the question of limited SNAP23 availability for LDs in fed state does not arise. Thus, feeding-fasting transitions induce appearance of two cholesterol binding proteins, flotillin-1 and SNAP23, in completely opposite manner to the LD membrane.

To investigate which cellular pathways recruit SNAP23 to LDs upon fasting, we first performed Western blotting of SNAP23 on PM-enriched fractions and ER-enriched microsome fractions prepared from liver of fed and fasted rats. No significant difference in SNAP23 was observed on the PM (Fig 4C) or on the microsomes (Fig 4D). Therefore, the PM and the ER may not be a major source for SNAP23 supply to FLDs. There is substantial evidence from cell culture studies that LD-mitochondria interactions increase after glucose starvation of cells, and this facilitates beta-oxidation using LD-derived TG in the mitochondria [25,28,29]. Indeed, we could detect abundant amounts of the mitochondrial membrane marker VDAC1 on FLDs, but VDAC1 could not be detected on NLDs (Fig 4E). This suggests that LD-mitochondria interactions are upregulated in fasted state in the liver. Such interactions may be mediated by increased SNAP23 on FLDs, and may serve to supply TG/FA to mitochondria for beta-oxidation when glucose levels are depleted after fasting. LD-mitochondria interactions are dramatically enhanced in muscle cells after exercise training of animals [42]. Knockdown of SNAP23 reduces LD-mitochondria association, eventually slowing down beta oxidation [25].

## Discussion

TG stored in liver in the form of cytosolic LDs increases massively after fasting because of FA influx into liver from adipose tissue [27]. Here we observed a significant reduction of flotillin-1 on LDs in the rat liver after fasting. Contrary to our finding, flotillin-1 increases on LDs when cultured cells are treated with FA [10]. This brings out important differences between a cell culture model and the *in vivo* scenario. Flotillin-1 can regulate activity of several kinases [17]. ERK2 and PKA are known kinases present on LDs [43], and could be possible targets for flotillin-1. The downstream consequence of differential flotillin-1 binding to LDs between fed and fasted states remains to be investigated.

**Fig 5:**
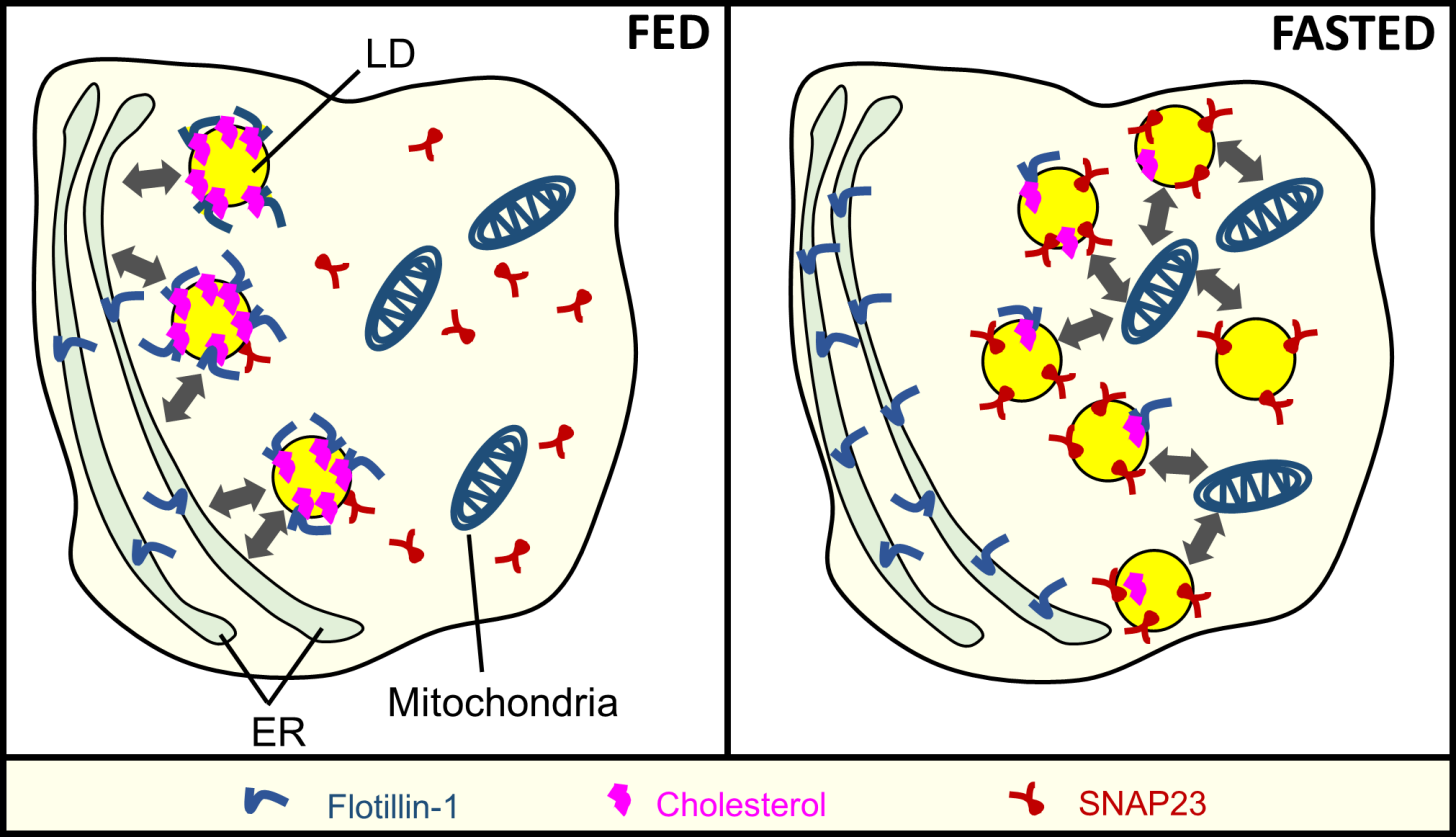
Working model for feeding-fasting dependent recruitment of microdomain associated proteins to lipid droplets in the liver. **Fed state:** Free cholesterol levels increase on the LDs, and may mediate ER-LD interactions (thick double headed arrows). Flotillin-1 is trafficked to LDs from ER, possibly because of enhanced ER-LD interactions. Cholesterol binding proteins (such as flotillin-1) therefore increase on LDs in the fed state. These proteins may reside on cholesterol induced microdomains on the LD membrane. **Fasted state:** There are more LDs in the liver due to increased flux of FA from adipose tissue into liver. However, these LDs appear to interact less with the ER. The reduced ER-LD interactions are evidenced by reduced transfer of GPAT4 to LDs from the ER (not shown in the schematic). Reduced ER-LD interactions may also block the transfer of flotillin-1 to LDs. However, proteins like SNAP23 which are supplied to LDs from sources other than ER (probably cytosol), are now abundant on the LD membrane. High levels of SNAP23 on LDs increase the interaction between LDs and mitochondria (grey arrows), as evidenced by presence of mitochondrial marker proteins on LDs (e.g. VDAC-1; not shown in the schematic).

Fig 5 presents a working model on the basis of our results. Flotillin-1 presence on ER-enriched microsomes showed a trend opposite to flotillin-1 presence on LDs, suggesting transfer of this protein from ER to LDs in fed state. Proteins that traffic from ER to LD share common topological features such as a hydrophobic motif, which allows their insertion in the ER membrane (3). Such motifs are known to exist on flotillin-1 and other proteins (e.g. stomatin) from the same family [18]. Interestingly stomatins are also recruited from ER to the LDs, though the significance of this is not well understood [9]. Since flotillin-1 has a well-studied role in cholesterol trafficking [44], cholesterol levels were measured on FLDs and NLDs. Though FC levels on LDs were very low (micrograms), we observed more cholesterol on the NLD membrane as compared to FLDs. Notably, a positive correlation between cholesterol and flotillin-1 recruitment was demonstrated by others and by us on phagosomes [35,45]. The possible trafficking of flotillin from ER to LDs could result from enhanced ER-LD interactions in the fed state of liver. This possibility was supported by observing more GPAT4 on NLDs (Fig 2C). Accumulation of FC on LDs in cultured hepatocytes promotes LD fusion with the ER [40]. Increased FC on LDs in the fed state of liver may therefore mediate ER-LD interactions, trafficking flotillin-1 and GPAT4 from ER to LDs.

Unlike flotillin-1, SNAP23 levels were higher on FLDs when compared to NLDs. This was unexpected, because SNAP23 is known to bind cholesterol [46] and FLDs have lesser FC than NLDs (Fig 2D). However, a requirement of cholesterol for SNARE/SNAP23 recruitment has only been discussed for bilayer membranes [23], and the mechanism for SNAP23 recruitment to the monolayer membrane of LDs could be independent of cholesterol. This issue has not been discussed in earlier reports of SNAP23 association with LDs [24,47]. Interestingly, the mitochondrial marker protein VDAC1 appeared on LDs during fasting (Fig 4E), suggesting that LD-mitochondria interactions increase in the liver in fasted state. There is substantial evidence that reduction in glucose increases LD-mitochondria interactions, so that FA-substrates can be provided to mitochondria for beta oxidation [28,29]. Further, LD–mitochondria interactions depend on SNAP23, and RNAi knockdown of SNAP23 prevents transfer of perilipin-2 from LDs to mitochondria, along with reduced beta oxidation in NIH3T3 cells [25]. Increased SNAP23 on FLDs could therefore facilitate FA supply to mitochondria from LDs. Both flotillin-1 and SNAP23 are highly dependent on post translational modifications like palmitoylation for binding the plasma membrane [10,23]. Differences in post translational modification of these proteins during feeding or fasting could regulate their binding affinity for the LD membrane. However, these issues remain to be addressed.

To summarize, we have reported the differential recruitment of two membrane-microdomain proteins to LDs in the liver in response to feeding/fasting cycles (Fig 5). LDs are dynamic organelles that exchange proteins/lipids with other cellular components in metabolically regulated manner [3,29]. Lipid microdomains and microdomain-associated proteins evoke special interest in this context as a platform for signal transduction, cell polarity, trafficking and pathogen entry [17]. It is possible that LDs serve different functions in fed and fasted states in the liver, and some of these functions are initiated by differential recruitment of microdomain-associated proteins to LDs in these two states. In this sense, LDs appear to be quite sensitive to metabolic fluctuations. The fasting-induced changes in flotillin-1, SNAP23 and FC on LDs observed here evoke interest in the role of LDs in metabolism-related pathological conditions. Notably, a large amount of free cholesterol was detected in adipocytes, cells that have a very large content of LDs [48]. The adipose tissue was therefore proposed to play a major role in clearance of cholesterol from plasma. Since the liver and adipose tissue are important for lipid/cholesterol homeostasis, understanding how cholesterol is trafficked to LDs in these tissues becomes important.

Our report is largely limited to end-state biochemical assays on LDs purified from liver, and cannot elucidate the kinetics of protein recruitment to LDs in liver. However, our studies do bring out physiologically relevant pathways for protein trafficking to LDs in the liver in response to feeding/fasting cycles. These pathways cannot be fully explored, and may not even reproduce in cultured cells because the required metabolic signals are absent. We hope that improved technologies for *in-vivo* imaging will permit better understanding of these phenomena and their spatio-temporal dynamics inside the liver in future. This should be a worthwhile effort, because inability of the liver to control lipid flux across the feeding-fasting cycles is a precursor to fatty liver conditions [49]. Further, several kinds of virus that target the liver (e.g. Hepatitis-C, Dengue) interact intimately with LDs, and may also require LDs to interact with the ER for viral replication [50]. Because microdomains on the plasma membrane function as “gates” for pathogen entry into cells [51], microdomains on LDs may have a role in viral replication.

## Materials and methods

### Reagents

Perilipin-2 antibody (651102) was purchased from Progen Biotechnik, Germany. GPAT4 antibody was from Abcam (Cat. #ab 76707). Antibodies against flotillin-1 (133497), SNAP23 (3340), Na^+^-K^+^ATPase (7671), VDAC1 (15895) and HSP60 (46798) were purchased from Abcam. Antibodies against PDI (610946) and Golgin-97(21270) were purchased from BD Biosciences (San Jose, CA) and Invitrogen respectively. Actin antibody (AANO1-A) was purchased from Cytoskeleton Inc. (Denver, CO). All the other reagents were purchased from Sigma-Aldrich, Bengaluru, India. Silica based TLC plates were from Merck. TG standards were from Avanti Polar Lipids, Inc. (Alabaster, Alabama).

### Animals

3–4 month old male Sprague Dawley rats were used for all the experiments. The procedures on animals were approved by the Institutional Animal Ethics Committee formulated by the Committee for the Purpose of Control and Supervision of Experiments on Animals (CPCSEA). Rats in the ‘Fed group’ were maintained on a regular 12-hour light/12-hour dark cycle, and fed on the standard laboratory diet. Rats in the ‘Fasted group’ were fasted for 16 hours. Water availability was not restricted for both groups of animals. Rats from the same litter were used for a fed-fasted pair.

### Isolation of LDs

LDs were isolated from the liver of animals using the sucrose step gradient method [33,34]. For this procedure animals were anaesthetised by sodium thiopentone injection. The anaesthetised animal was dissected and perfusion of liver was carried out through hepatic portal vein. The liver was perfused with approximately 30ml of 1XPBS. The tissue was then chopped into fine pieces and homogenised using a Dounce homogeniser in homogenisation buffer (35mM PIPES, 5mM EGTA, 5mM MgSO_4_ and 1M Sucrose pH-7.2). Protease inhibitor cocktail (2x), 4mM DTT, 4mM PMSF and 2μg/ml of pepstatin were added to the buffer before homogenisation to inhibit protease activity. All the steps including homogenisation were carried out at 4°C. After homogenisation, a PNS (post nuclear supernatant) was prepared by centrifuging the tissue homogenate at 1800*g* at 4°C for 10 minutes. The supernatant (PNS) was collected without disturbing the pellet. This liver PNS fraction was then mixed with 2.5M sucrose buffer to a final molarity of 1.6M, and loaded at the bottom of a sucrose step gradient in a SW32 rotor. This layer was overlaid with 1.4M, 1.2M, 0.5M and 0M sucrose, all prepared by mixing sucrose in the homogenisation buffer. The sucrose gradient was spun at 110000x*g* at 4°C for 1 hour. LD fraction was collected from the top of the gradient and immediately concentrated by centrifugation (14000*g* for 10 minutes at 4°C). This leads to accumulation of buoyant LDs at the top layer. Next, the infranatant is carefully removed with the help of a syringe and the top LD layer is allowed to float in a small volume of 500μl of the same buffer. This procedure for concentration of the LD fraction was required for all the western blotting related experiments in order to minimise the volume of LD sample to be loaded in SDS-PAGE gels. Later, the LD fraction was resuspended using a pipette with a wide bore tip. This step was simultaneously performed for both NLD and FLD fraction to maintain uniformity of the procedure. The NLD and FLD samples were then normalised for TG amount (see next section), aliquoted and stored at −20°C for further use.

### LD normalisation and thin layer chromatography

NLD and FLD samples were adjusted to obtain equal value of light scattering (as measured by optical density at 400nm (OD_400_) for both the samples in a spectrophotometer. Such “normalised” NLD and FLD samples were also observed to have equal TG amounts, as measured in a TLC experiment. The sensitivity of this method was estimated by serially diluting a LD sample and plotting OD_400_ against the TG band intensities obtained by TLC. A linear variation was observed (not shown).

For TLC, lipids were extracted from samples using the standard procedure described [52]. After extraction, the lipid samples were resuspended in chloroform and spotted on silica TLC plates (Merck) using a glass capillary. The silica plates were pre-run in chloroform and air-dried before spotting of the samples. Two solvent systems were used to resolve TG [38]. First, the plates were run partially in hexane:diethyl ether:acetic acid in the ratio 70:30:1. The plates were then removed from the TLC chamber and air dried. The second solvent system was hexane:diethyl ether in the ratio 49:1. This solvent was allowed to run close to the top of the plate. Next, the plate was air dried and sprayed with 10% CuSo_4_ in 8% H_3_PO_4_. After the solution had evaporated, the plate was baked in a hot air oven at 200°C for 15–20 minutes. TG and FC standards respectively were used to identify TG and FC in the samples. The image of the TLC plate was taken under white light illumination in a BioRad instrument. Intensity measurement of the TG and FC bands was done using ImageJ software.

### Liver tissue extract (TE) preparation

After perfusion, the liver was dissected and minced into small pieces in homogenisation buffer, followed by homogenisation in cold. The homogenate was centrifuged at 1000*xg* for 2min to remove large pieces of tissue. After centrifugation the tissue extract (TE) is recovered as supernatant, aliquoted and stored at −20°C. For depletion of LDs from TE, the TE was centrifuged at 500,000*xg* for 1hour. The top layer of LDs was carefully removed. The remaining part (containing a pellet and infranatant) was again resuspended in the infranatant and this sample was processed for western blotting.

### Microchamber immunofluorescence assay

This method has been adapted from [53] with modifications. A microchamber was prepared using a coverslip attached to glass slides with double stick tape. The LD sample (30μl) was then aspirated into the microchamber. Buoyant LDs were made to float and bind to the surface of coverslips by incubating the microchambers in a humidified box for 20 minutes (kept inverted with the coverslip on the upper side). In the next step 30μl of blocking solution (6% BSA in 1XPBS) was passed through the microchamber and incubation was done for 30 minutes. After incubation the microchamber was washed by passing 3x30μl of 1XPBS solution. Primary antibody solution (30μl) was prepared in blocking buffer and was aspirated into the microchamber. Incubation in primary antibody was done for 30 minutes followed by wash with 3x30μl 1XPBS solution. Next 30μl of secondary antibody dilution was aspirated in, and incubation was done for 30 minutes. Another wash with 3x30μl of 1XPBS was given to remove any nonspecific antibody binding. Imaging was done immediately to avoid bleaching of the signal. Primary antibodies for flotillin-1 was used at 1:50 dilution. Alexa 555 conjugated secondary antibody was used at 1:250 dilution.

### Free cholesterol (FC) estimation on LD sample

FC values were estimated on LD samples using an HDL and LDL/VLDL cholesterol assay kit (Abcam ab65390). The recommended protocol fluorimetric detection of FC was followed. This procedure detects only FC, and thus interference from cholesteryl esters is not expected. For this assay, total lipids were extracted from NLD and FLD sample followed by resuspension in the assay buffer provided in the kit. Dilutions and duplicate samples were prepared from this solution of total lipids in assay buffer. The reading for all the LD samples is an average of duplicate values.

### Isolation of microsomes from rat liver

ER-enriched microsomes were isolated from liver following a published protocol [54]. Liver tissue was chopped into fine pieces and homogenised in a Dounce homogeniser by 20 strokes. The homogenisation buffer was composed of 20mM Tris-HCl, 250mM sucrose and 1mM EDTA, pH 7.4 and was pre cooled to 4°C. The liver homogenate was then centrifuged at 500*xg* for 15 minutes to remove cell debris. The supernatant was collected and centrifuged again at 14000*xg* for 10 minutes to pellet down mitochondria. This step was repeated three times. The supernatant was then centrifuged at 106,000*xg* for 1 hour at 4°C. The resulting supernatant was removed and microsomes were collected as a pellet. Microsomes recovered from this step were given a high-salt wash to remove peripherally adhered proteins and LDs. The high-salt wash was done by resuspending the microsome fraction in a buffer composed of 0.5M KCl in 10mM Tris-HCl (with gentle homogenisation using a Dounce homogeniser). The microsomes were incubated in this buffer for 30 minutes at 4°C on a low speed shaker. Microsomes were again pelleted at 106,000*xg* for 1 hour. The microsome pellet was finally resuspended in 500μl of buffer containing 10mM Tris-HCl, pH 7.5 and 250mM Sucrose, aliquoted and stored at -80°C.

### Isolation of plasma membrane enriched fraction from rat liver

The plasma membrane (PM) fraction was isolated from rat liver according to a published protocol [55] with some modifications. Rat liver was dissected and homogenised in three volumes of homogenisation buffer composed of 20mM Tris-HCl, 250mM sucrose and 1mM EDTA, pH-7.4. For this report only the nuclear fraction (N) was used for isolation of PM. The nuclear fraction (N) was given two washes with the homogenisation buffer. PM was isolated as N2 fraction from the gradient as mentioned in the original protocol. The PM fraction (N2) was pelleted and washed once with 10mM Tris-HCL buffer with 250mM sucrose. After pelleting PM fraction was resuspended in the above buffer and stored at -80°C.

### Isolation of mitochondria from rat liver

The mitochondria fraction was isolated from rat liver by following the published protocol [25].

### Estimation of FC in rat liver extract, plasma membranes and LDs

Our purpose was not to estimate exact levels of FC, but just to obtain an approximate relative estimate between FC amount in liver and that on LDs. Crude liver tissue extract (TE) was prepared from a known mass of liver tissue. A TLC experiment was run with this TE and known dilutions of FC as standards (not shown). The best match was used to estimate total amount of FC in the sample (*N* = 3 rats used). Similarly, PM and LDs were prepared and comparison was done with FC standards in a TLC experiment. The total FC on PM and LDs was then estimated for the whole liver (by multiplication with the Ratio (Weight of the whole Liver / Weight of Liver used in sample).

### Western blots

For western blotting of NLD and FLD samples, equal volume of “normalised samples” (see earlier) was loaded on a 10% SDS-PAGE gel. Overnight transfer of the proteins was done at 45mA on a PVDF membrane (Roche) at room temperature. A similar procedure was adapted for western blotting of TE and ER-microsome samples. Protein estimation of TE and ER samples was done using a BCA kit. After overnight transfer of proteins on PVDF membrane, the membrane was blocked using 5% dried milk prepared in 0.1% Tween-PBS. Primary antibody dilutions were prepared in blocking solution. After incubation with primary antibodies, membranes were washed thrice with 0.1% Tween-PBS and incubated with peroxidase-conjugated secondary antibodies (1:10,000 Santa Cruz Biotechnology) and detected with ECL reagent (WBKLS0500, Millipore) on X-ray films.

### Immunohistochemistry (IHC) of liver tissue

IHC on liver sections was done following a published protocol [56]. Whole body perfusion of anaesthetised animals was done using normal saline for 15 minutes, followed by 4% PFA, till full body twitching and clearance of the tissue was observed. Perfusion of 4% PFA was carried out for around 20 minutes, after which the liver was dissected out, cut into small pieces of 3–4 inches and stored in 40% sucrose solution prepared in 1XPBS at 4°C for around 36 hours to allow complete sinking of the tissue pieces in the buffer. Next, sectioning of tissue pieces was carried out in a cryostat (Leica Biosystems). Tissue pieces were mounted in OCT solution (Fisher Scientific) and 10μm thick sections were acquired in the cryostat machine. Superfrost slides (Thermo Scientific) were used for placing the sections. The sections were fixed again in 4% PFA for 10 mins followed by three with 1xPBS for 10 mins each. Antigen retrieval was performed on the sections before incubation with the primary antibody. The composition of the antigen retrieval buffer was 10mM Sodium citrate, 0.05% tween 20, pH 6.0. Sections were incubated in antigen retrieval buffer in a boiling water bath at 98°C for 3–4 minutes. Sections were washed once with 1XPBS and then blocking was performed in 3% BSA solution in 1XPBS for 1hour. Sections were then washed with 1XPBS and incubated in primary antibody solution for 1 hour at room temperature. The sections were then washed thrice with 1XPBS, 10 minutes each. Incubation in secondary antibody solution was also done for 1hour at room temperature followed by three washes of 10 minutes each with 1XPBS. After antibody incubations, sections were mounted with Vectashield (Vectorlabs) and imaged on a confocal microscope.

### Confocal microscopy

Imaging of all the samples was done on a Zeiss LSM 710 confocal microscope with 63x oil objective.

### Statistical analysis

All experiments used three pairs of rats (fed and fasted) unless otherwise stated. Statistical analysis was done to determine *P*-values by the t-test method (one-sample or two-sample t-test, as relevant).
